# A Retrospective Cohort Study on Maternal and Neonatal Clinical Characteristics and Outcomes of COVID-19: Does the Gestational Age Affect the Outcome?

**DOI:** 10.7759/cureus.35188

**Published:** 2023-02-19

**Authors:** Esra Ozbasli, Selin Ozaltin, Elif G Aygun, Nazli Albayrak, Ozguc Takmaz, Faruk S Dede, Mete Gungor

**Affiliations:** 1 Obstetrics and Gynecology, Acıbadem University, Istanbul, TUR; 2 Obstetrics and Gynecology, Acıbadem University Hospital Atakent, Istanbul, TUR

**Keywords:** neonatal mortality, maternal mortality, pandemic, pregnancy, covid-19, sars-cov-2

## Abstract

Background

To evaluate the maternal and neonatal clinical characteristics and outcomes of COVID-19 during pregnancy and to see whether infection with COVID-19 before or after the 20th gestational week affects these outcomes.

Methods

We conducted a retrospective study with data from pregnant women who were followed up and delivered at Acibadem Maslak Hospital between April 2020 and December 2021. Their demographics and clinical data were reviewed and compared.

Results

Among 1223 pregnant women, 42 (3.4%) were diagnosed with COVID-19 (SARS-CoV-2-positive). Approximately 52.4% of the 42 pregnant women with COVID-19 were diagnosed during or before the 20th gestational week, while 47.6% were positive after the 20th gestational week. The preterm birth rate was 11.9% and 5.9% in infected and uninfected pregnant women, respectively (p>0.05). In the infected pregnant women, the rate of preterm rupture of membranes (PROM) was 2.4%, small for gestational age (SGA) was 7.1%, cesarean delivery was 76.2%, and neonatal intensive care unit (NICU) admission was 9.5%. These rates among uninfected women were 0.9%, 9.1%, 61.7%, and 4.1%, respectively (p>0.05). Maternal ICU admission and intrapartum complications were higher in infected pregnant women (p>0.05). Postpartum hemorrhage (PPH), intrauterine growth retardation (IUGR), neonatal infection, and fetal demise were absent in SARS-CoV-2-positive pregnant women. Having a high school or lower education level significantly increased the risk of SARS-CoV-2 infection during pregnancy 10 times. Also, a one-week increase in gestational age significantly reduced the risk of SARS-CoV-2 infection during pregnancy. When SARS-CoV-2-positive pregnant women were compared according to whether or not they were positive before or after the 20th gestational week, there was no statistically significant difference between the two groups in terms of maternal outcomes, neonatal outcomes, and demographic characteristics.

Conclusions

COVID-19 during pregnancy did not adversely affect maternal and neonatal outcomes. Also, whether pregnant women were infected before or after the 20th gestational week did not have a negative impact on maternal and neonatal outcomes. However, infected pregnant women should be followed closely, and they should be informed in detail about the possible adverse outcomes and the importance of precautions for COVID-19.

## Introduction

COVID-19, caused by SARS-CoV-2, has been a life-threatening international health emergency since the first case was detected in December 2019 [1]. According to data shared by the Center for Systems Science and Engineering (CSSE) at John Hopkins (https://coronavirus.jhu.edu/map.html), the total number of cases of COVID-19 disease was 618 million worldwide as of October 03, 2022. Due to physiological changes, immaturity, and immunosuppression, pregnant women and their babies are in the high-risk group [2]. Because of these changes, the response to COVID-19 infection may be more severe during pregnancy. According to the weekly report published by the American CDC in June 2020, 31.5% of pregnant women with COVID-19 were hospitalized, while this rate was 5.8% in non-pregnant women. In addition, it has been reported that the rates of hospitalization in the ICU and the need for mechanical ventilation were significantly higher in pregnant women [3]. In a meta-analysis conducted in 2021, it was shown that the rates of preterm birth (PB), preeclampsia (PE), stillbirth, admission to the maternal ICU, neonatal low birth weight (LBW), and neonatal intensive care unit (NICU) hospitalization were higher in those who had COVID-19 during pregnancy [4-6]. This situation highlights how important it is for pregnant women and their relatives to take precautions against the transmission of COVID-19. Although studies on maternal and neonatal outcomes of COVID-19 in pregnancy are limited, most compare pregnant and non-pregnant women with the disease [7,8]. Furthermore, the number of studies comparing outcomes according to gestational age is limited. Most of the studies evaluating the maternal and neonatal outcomes of COVID-19-positive pregnant women include pregnant women who are diagnosed with COVID-19 during the third trimester or during hospitalization for delivery [9-11]. The number of studies evaluating the outcomes of pregnant women infected during the first or second trimester is also limited [12,13]. This study aimed to compare maternal and fetal outcomes among pregnant women with COVID-19 and assess whether differences in outcomes differ by gestation age.

## Materials and methods

A total of 1738 pregnant women who gave birth at Acibadem Maslak Hospital between April 2020 and December 2021 and underwent PCR testing for SARS-CoV-2 performed on nasopharyngeal swab samples during their pregnancy or on admission to the hospital were included in the study. We excluded 366 of these patients from the study because they were followed up in another clinic and 149 due to a lack of data in their files or multiple pregnancies. In the end, we included 1223 patients in the study. The files of these patients were reviewed retrospectively. In addition, the patients were called and questioned on their symptoms during the COVID-19 disease, the medicines they used, and their vaccination statuses. Our hospital is a private university hospital where most of the patients with high socio-cultural levels are followed up. These pregnant women were compared in terms of data which included maternal demographics, medication during pregnancy, the gestational week when COVID-19 was diagnosed, antenatal diseases occurring after COVID-19 detection during pregnancy, intrauterine growth retardation (IUGR), small for gestational age (SGA), fetal distress, intrauterine fetal demise (IUFD), intrapartum complications, gestational age (GA) at delivery, mode of delivery, postpartum hemorrhage (PPH), admission to the maternal ICU, birth weight, admission to the NICU, and the indication for NICU hospitalization. Since the beginning of the pandemic, all hospitalized patients, regardless of exposure history or symptoms, are routinely tested for COVID-19 via PCR in our hospital. In addition, patients with symptoms suggestive of COVID-19 during pregnancy also underwent PCR testing. While those with a positive PCR test and no COVID-19 symptoms were considered asymptomatic, symptomatic COVID-19 cases were divided into groups according to the presence of fever, cough, myalgia, loss of taste and smell, and flu-like symptoms. COVID-19-positive pregnant women were referred to the infectious diseases department. Low-molecular-weight (anti-Xa IU/0.4 mL) heparin injection was administered to patients for whom the infectious diseases physician recommended anticoagulant use as a result of clinical and laboratory evaluations. In addition, the data of patients who were SARS-CoV-2-positive at ≤20 weeks of gestation and those who were positive at >20 weeks of gestation were compared to see if the gestational age affects the patient’s clinical characteristics, maternal outcome, and neonatal outcome. The study was approved by the Medical Ethics Committee of the Institutional Ethical Review Board of the Acıbadem Mehmet Ali Aydınlar University School of Medicine on February 14, 2022 (ATADEK-2022-03/02).

Statistical analysis

Continuous variables were expressed as the mean ± SD and/or the median (min-max), while categorical data were expressed as frequencies and percentages. Normality analyses for continuous variables were performed using the Kolmogorov-Smirnov Goodness-of-Fit test. Student’s t-test was used for comparisons of normally distributed data between the two groups, while the Mann-Whitney U test was used in performing such comparisons for skewed continuous data. Categorical data were compared using the chi-square test and Fisher’s exact test. Using the possible factors identified in previous analyses in predicting risk in SARS-CoV-2-positive women, independent predictors were identified using univariate logistic regression analyses (enter method). Variables that were significant (p <0.25) in the univariate analyses qualified for the multivariate model and underwent multiple logistic regression analyses (Backward LR). The Hosmer and Lemeshow test, Omnibus tests of model coefficient, and Nagelkerke R Square values were given for model fit and significance. All statistical analyses were performed with IBM SPSS version 26.0 (IBM Corporation, Armonk, NY, USA), and the threshold for statistical significance was set at p <0.05.

## Results

Within the scope of this study, 42 (3.4%) of 1223 pregnant women who applied to the Gynecology and Obstetrics department of Acıbadem Maslak University Hospital for routine pregnancy follow-up or delivery between April 1, 2020, and December 31, 2021, were SARS-CoV-2-positive (diagnosed with COVID-19).

Sociodemographic and clinical characteristics

Although statistically insignificant, the mean age of SARS-CoV-2-positive pregnant women (35.43±4.33) was higher than that of SARS-CoV-2-negative pregnant women (34.28±4.54) (p = 0.066). All pregnant women in our study were Caucasian. When BMI values, parity, neonatal gender, neonatal infection, maternal disease, and antenatal medication use were searched, we found no statistically significant difference between SARS-CoV-2-positive (COVID-19 positive) and COVID-19-negative pregnant women. As for the educational status, we found that 92.9% of infected women and 99.2% of COVID-19-positive women were university graduates, and the difference was statistically significant (p < 0.001) (Table 1). 

**Table 1:** Sociodemographics and clinical characteristics.

		SARS-CoV-2-positive pregnant women (n=42)	SARS-CoV-2-negative pregnant women (n=1181)	P-value
Age (year) (Mean±SD)		35.43±4.33	34.28±4.54	0.066***
BMI (kg/m^2^) (n%)	<25	10 (23.8%)	349 (29.6%)	0.448*
	25-29.9	26 (61.9%)	567(48%)	
	30-34.9	4 (9.5%)	202 (17.1%)	
	35-39.9	2 (4.8%)	56 (4.7%)	
	≥40	0 (0%)	7 (0.6%)	
Educational status (n%)	University	39 (92.9%)	1172 (99.2%)	<0.001*
	High school	2 (4.8%)	9 (0.8%)	
	Middle school	1 (2.4%)	0 (0%)	
Smoking (n%)	Non-smoker	42 (100%)	1172 (%99.2)	1.000**
	Smoker	0 (0%)	9 (0.8%)	
Alcohol use (n%)	No	42 (100%)	1165 (98.6%)	1.000**
	Yes	0 (0%)	16 (1.4%)	
Antenatal disease (n%)	No	40 (95.2%)	1144 (96.9%)	0.391**
	Yes	2 (4.8%)	37 (3.1%)	
COVID-19 symptoms (n%)	Asymptomatic	4 (9.5%)	-	-
	Fever	5 (11.9%)	-	-
	Cough	2 (4.8%)	-	-
	Loss of taste/smell	2 (4.8%)	-	-
	Flu-like	18 (42.9%)	-	-
	Myalgia	11 (26.2%)	-	-
Treatment protocol (n%)	No medicine	27 (64.3%)	-	-
	Paracetamol	13 (31%)	-	-
	Favipiravir	2 (4.8%)	-	-
Use of anticoagulant (n%)	No	31 (73.8%)	-	-
	Yes	11 (26.2%)	-	-
COVID-19 vaccine during pregnancy (n%)	No	10 (23.8%)	-	-
	Yes	32 (76.2%)	-	-
Gestational age at COVID-19 disease (n%)	≤20th week	22 (52.4%)	-	-
	>20th week	20 (47.6%)	-	-
*Chi-square test **Fisher’s Exact test ***Mann Whitney U Test				

The incidence of PE (2.4%) and gestational diabetes (GDM) (2.4%) in COVID-19-positive pregnant women were found to be higher than in COVID-19-negative patients (1% and 1.5%, respectively) (p = 0.367 and p = 1.000, respectively). In addition, while cholestasis was not observed in infected pregnant women, it occurred in 0.6% of COVID-19-negative pregnant women. 

Maternal and neonatal outcomes

Comorbidities were observed in 14.3% of infected pregnant women. Five of them had hypothyroidism, and one had diabetes mellitus. The mean gestational age and the mean weight of the neonatal of COVID-19-positive pregnant women at birth were lower than those of COVID-19-negative pregnant women (p = 0.111 and p = 0.641. respectively). The CS rate in COVID-19-positive pregnant women (76.2%) was higher than that in negative pregnant women (61.7%), and the difference was almost statistically significant (p = 0.057). The rate of PB was higher in COVID-19-positive pregnant women (11.9%) than in negative ones (5.9%) (p = 0.176). IUGR or fetal demise was not observed in any COVID-19-positive pregnant women. While intrapartum complications were not observed in any of the COVID-19-negative pregnant women, they occurred in 2.4% of COVID-19-positive pregnant women, and the difference in their rates was statistically significant (p = 0.034). As an intrapartum complication, fever was observed in one of the COVID-19-positive pregnant women. In our study, it was observed that the rate of hospitalization in the maternal ICU was 2.4% among COVID-19-positive women, and this was statistically significant (p = 0.034). Although statistically insignificant, postpartum complication rates were higher in COVID-19-positive pregnant women than in COVID-19-negative ones (2.4% and 0.9%, respectively; p = 0.344). Surgical site infection was observed in one of the COVID-19-positive pregnant women as a postoperative complication. It was determined that none of the pregnant women examined within the scope of the study had PPH. The rate of NICU admission was higher in COVID-19-positive pregnant women (9.5%) (p = 0.104). Among the neonates of COVID-19 positive, four were hospitalized in the NICU, and the indication for hospitalization in all of them was prematurity. Maternal and neonatal clinical characteristics and outcomes are summarized in Table 2.

**Table 2:** Maternal and neonatal clinical characteristics and outcomes. NICU: Neonatal intensive care unit.

		SARS-CoV-2-positive pregnant women (n=42)	SARS-CoV-2-negative pregnant women (n=1181)	P-value
Birth weight (g) (Mean±SD)		3247.5±656.76	3284±1117.39	0.641***
Gestational age at delivery (Mean±SD)		37.83±2.34	38.57±1.38	0.111***
		(n%)	(n%)	
Gestational age during COVID-19 disease (Mean±SD)		19.98±7.85	-	-
Parity	Nulli parity	31 (73.8%)	911 (77.1%)	0.614*
	Multi parity	11 (26.2%)	270 (22.9%)	
Mode of delivery	Spontaneous vaginal	10 (23.8%)	452 (38.3%)	0.057*
	Cesarean delivery	32 (76.2%)	729 (61.7%)	
Preterm birth (<37 weeks)	No	37 (88.1%)	1111 (94.1%)	0.176**
	Yes	5 (11.9%)	70 (5.9%)	
Neonatal sex n (%)	Female	18 (42.9%)	563 (47.7%)	0.539*
	Male	24 (57.1%)	618 (52.3%)	
Maternal comorbidity	No	36 (85.7%)	934 (79.1%)	0.297*
	Yes	6 (14.3%)	247 (20.9%)	
Antenatal medicine use	No	36 (85.7%)	931 (78.8%)	0.281*
	Yes	6 (14.3%)	250 (21.2%)	
Postoperative complication	No	41 (97.6%)	1170 (99.1%)	0.344**
	Yes	1 (2.4%)	11 (0.9%)	
Premature rupture of membranes (PROM)	No	41 (97.6%)	1144 (96.9%)	1.000**
	Yes	1 (2.4%)	37 (3.1%)	
Fetal complications	No	39 (92.9%)	1058 (89.6%)	0.798**
	Yes	3 (7.1%)	123 (10.4%	
Intrapartum complication	No	41 (97.6%)	1181 (100%)	0.034**
	Yes	1 (2.4%)	0 (0%)	
Maternal ICU admission	No	41 (97.6%)	1181 (100%)	0.034**
	Yes	1 (2.4%)	0 (0%)	
Neonatal infection	No	42 (100%)	1132 (95.9%)	0.409**
	Yes	0 (0%)	49 (4.1%)	
NICU admission	No	38 (90.5%)	1132 (95.9%)	0.104**
	Yes	4 (9.5%)	49 (4.1%)	
* Chi-square Test **Fisher's Exact Test ***Mann-Whitney U Test				

SARS-CoV-2-positive pregnant women

COVID-19-positive women were also compared in terms of demographic features and clinical features by dividing them into two groups: those who were positive at ≤20 weeks of gestation and those who were positive at >20 weeks of gestation. Among 42 pregnant women with COVID-19, 22 (52.4%) were positive at ≤20 weeks of gestation. Twenty of them (47.6%) were positive at >20 weeks of gestation. Among COVID-19-positive women, the mean gestational age at COVID-19 diagnosis was 19.98±7.85 weeks. It was observed that none of the COVID-19-positive women used alcohol or smoked. Also, there was no statistically significant difference in demographic characteristics between COVID-19-positive and negative pregnant women. The gestational age at birth was higher in those who were positive at ≤20 weeks of gestation (38.35±1.45 weeks) than those who were positive at >20 weeks of gestation (37.27±2.97 weeks) (p = 0.154). Considering the mode of delivery, the rate of CS was higher in pregnant women who were infected at >20 weeks of gestation (85%). When the two groups were compared in terms of PB, it was observed that this rate was higher in those who were infected at >20 weeks of gestation (20%) (p = 0.174). IUGR, fetal demise, PPH, maternal mortality, and neonatal infection were not observed in any of the patients who were COVID-19-positive during pregnancy. No statistically significant difference was observed between the two groups in terms of the neonate’s sex, neonate’s birth weight, the presence of antenatal disease, antenatal medicine use, COVID-19 symptoms, anticoagulant use, postoperative complications, PROM, SGA, intrapartum complications, and ICU hospitalization rate (Table 3).

**Table 3:** Comparison of clinical characteristics according to the gestational age during COVID-19 disease.

		Gestational age during COVID-19 disease		
		≤20th week (n=22)	>20th week (n=20)	P-value
Gestational week (mean±SD)		38.35±1.45	37.27±2.97	0.154***
Birth weight (g) (mean±SD)		3285±384.59	3206.25±874.01	0.713***
		(n.%)	(n.%)	
Parity	Null parity	17 (77.3%)	14 (70%)	0.592*
	Multi parity	5 (22.7%)	6 (30%)	
Mode of delivery	Spontaneous vaginal	7 (31.8%)	3 (15%)	0.201*
	Cesarean delivery	15 (68.2%)	17 (85%)	
Preterm birth (<37 weeks)	No	21 (95.5%)	16 (80%)	0.174**
	Yes	1 (4.5%)	4 (20%)	
Neonate sex n (%)	Female	11 (50%)	7 (35%)	0.327*
	Male	11 (50%)	13 (65%)	
Maternal comorbidity	No	21 (95.5%)	15 (75%)	0.087**
	Yes	1 (4.5%)	5 (25%)	
Antenatal medicine use	No	21 (95.5%)	15 (75%)	0.087**
	Yes	1 (4.5%)	5 (25%)	
COVID-19 symptoms	Asymptomatic	2 (9.1%)	2 (10%)	0.788*
	Fever	2 (9.1%)	3 (15%)	
	Cough	1 (4.5%)	1 (5%)	
	Loss of taste/smell	2 (9.1%)	0 (0%)	
	Flu-like	10 (45.5%)	8 (40%)	
	Myalgia	5 (22.7%)	6 (30%)	
COVID-19 treatment protocol	No	16 (72.7%)	11 (55%)	0.233*
	Paracetamol	6 (27.3%)	7 (35%)	
	Favipiravir	0 (0%)	2 (10%)	
Use of anticoagulant	No	19 (86.4%)	12 (60%)	0.052*
	Yes	3 (13.6%)	8 (40%)	
Postoperative complications	No	21 (95.5%)	20 (100%)	1.000**
	Yes	1 (4.5%)	0 (0%)	
PROM	No	22 (100%)	19 (95%)	0.476**
	Yes	0 (0%)	1 (5%)	
SGA	No	20 (90.9%)	19 (95%)	1.000**
	Yes	2 (9.1%)	1 (5%)	
Intrapartum complication	No	22 (100%)	19 (95%)	0.476**
	Yes	0 (0%)	1 (5%)	
Maternal ICU admission	No	22 (100%)	19 (95%)	0.476**
	Yes	0 (0%)	1 (5%)	
NICU admission	No	21 (95.5%)	17 (85%)	0.333**
	Yes	1 (4.5%)	3 (15%)	
COVID-19 vaccine during pregnancy	No	8 (36.4%)	2 (10%)	0.071**
	Yes	14 (63.6%)	18 (90%)	
* Chi-square Test **Fisher's Exact Test ***Student's T-test				

According to the univariate logistic regression analysis to determine the factors that increase the risk of COVID-19 positivity in pregnancy, taking being a university graduate as a reference, having a high school or lower education level significantly increased the risk 10 times (OR: 10.017; 95% CI: 2.610-38.448), whereas a one-week increase in the gestational age significantly reduced the risk (OR: 0.796, 95% CI: 0.690-0.918) (p = 0.002 and p = 0.001, respectively). In the multivariate multiple logistic regression analysis where the variables of maternal age, gestational age at delivery, educational status, BMI, mode of delivery, PB, and NICU admission were included in the model, again, the gestational age and educational status remained significant in the model (p = 0.001 and p = 0.001, respectively).

## Discussion

In our study, we evaluated the maternal and neonatal clinical characteristics and outcomes of COVID-19 during pregnancy and investigated whether the gestational age affected these results. We believe that measures taken to protect public health, such as wearing masks, social distancing, and lockdowns during the pandemic, effectively reduced the rates of SARS-CoV-2 transmission, which could explain the low number of SARS-CoV-2-positive pregnant women in the early period. Also, we believe the fact that most of the pregnant women included in our study were university graduates and individuals with a high socio-cultural level contributed to their taking the necessary precautions and being careful in the follow-up of pregnancy and that it contributed to the low infection rates in our clinic. In Figueiro-Filho EA et al.'s study among pregnant women, 0.07% were infected with COVİD-19, but the infected pregnant ratio is believed to be much higher than this [14]. In our study, this ratio was higher (3.4%). In Ahlberg M et al.' study [15], the rate of education for >12 years in COVID-19-positive women was 35.5% [16-18]. In our study, this rate was much higher. Also, in our study, in line with the literature, it was shown that having a high school, or lower education level increased the rate of COVID-19 positivity during pregnancy 10 times. The mean maternal age in our study is similar to the findings of previous studies [8,19]. In our study, the most common symptoms in COVID-19-positive pregnant women were flu-like symptoms (ache, fever, headache, nasal congestion, and a runny nose) and myalgia, while in Khoury R et al.'s study, cough (22.4%) and fever (19.1%) were the most common [20]. The CDC reported that among infected pregnant women, the most common symptoms were cough (>50%) and shortness of breath (30%) [3]. In a meta-analysis, the loss of taste and smell were observed in 16.9% and 27.9%, respectively, in COVID-19-positive pregnant women; however, this rate was found to be lower in our study (4.8%) [14]. While the proportion of asymptomatic patients ranged from 2.9% to 42.3% in this meta-analysis, it was found to be 9.5% in our study, similar to Elshafeey F et al.'s study, which reported that 7.5% of patients were asymptomatic; conversely, Adhikari EH et al.'s cohort study reported 92% of asymptomatic patients [11,21].

Consistent with the findings of previous studies, the rate of CS was found to be high in our study [8,14,22]. The common view of most studies is that COVID-19 should not be an indication for delivery or CS. The timing and mode of delivery should be decided according to obstetric indications and maternal condition. In a prospective cohort study in which 241 COVID-19-positive pregnant women were evaluated, the rate of PB was 14.6%, similar to that in our study [20]. In Di Mascio D et al.'s meta-analysis, the rate of PE was 14.6%, although similar to Zhang L et al.'s study, this rate was lower in our study (2.4%) [8,23]. In Ahlberg M et al.'s research letter, as in our study, no significant difference was observed between COVID-19-positive and negative pregnant women in terms of PPH, route of delivery, PB, and birth weight [15]. In Figueiro-Filho EA et al.'s review, the rate of admission to the NICU was 20%; however, it was lower in our study [14]. While fetal demise was not observed in any infected pregnant woman in our study, it occurred in 0.6% of women in the research letter authored by Ahlberg M et al. and 1.7% in the review authored by Figueiro-Filho EA et al. [14,15]. However, the low number of COVID-19 patients in our study may have caused a lower incidence of rare complications such as fetal demise. In our study, the rate of admission to the maternal ICU among COVID-19 positive was similar to the rates reported in some previous studies [11,14]. The CDC report stated increased admission rates to the maternal ICU [16]. In a study by Zaigham M and Andersson O, this rate was observed as 3%. It has been reported that pregnant patients with severe COVID-19 are more likely to require ICU admission [2]. While it has been shown that the maternal mortality rate ranged from 0% to 11.1% in some reviews, no maternal death was observed in our study [2,20,24]. The maternal mortality rate was stated as 0.09% in the report from the CDC [3]. In a review, maternal and neonatal outcomes were not worse and did not differ from those in the general population, as stated in our study [14]. However, a review conducted in 2021 suggests that COVID-19 in pregnancy increases the risk of PE, stillbirth, PB, and NICU admissions [4]. In another study, it is stated that the odds of adverse maternal outcomes were 3.4 times higher. In contrast, the odds of adverse neonatal outcomes were 1.7 times higher among cases than controls [6]. However, in our study, these rates did not differ significantly between COVID-19-positive and negative pregnant women.

Khoury R et al.'s study [20] looked at whether there was an association between the severity of COVID-19 and the duration of pregnancy and found no relationship. In our study, no significant difference was observed between maternal and neonatal outcomes of pregnant women who had COVID-19 before the 20th gestational week and those who had it after 20 weeks of gestation. Data on COVID-19 prognosis and treatment modalities in the early period of the pandemic, especially in pregnant women, were limited. For this reason, it is striking that pregnant women avoided taking medication, especially during the first trimester. In our study, it was observed that while the use of drugs during the infection was 27.3% before the 20th gestational week, it increased to 45% after the 20th gestational week. It has been observed that the prognosis is worse in severely ill patients with coagulopathy, and it has been demonstrated that the prognosis in terms of mortality is better in patients using LMWH. Also, the anti-inflammatory properties of LMWH can be beneficial in COVID-19 [25]. In our study, the overall rate of anticoagulant use in COVID-19-positive pregnant women was 26.2%. The anticoagulant use rate was lower before 20 weeks of gestation. The strength of our study is that it compared the clinical characteristics, maternal outcomes, and neonatal outcomes according to gestational age. It evaluated not only infected pregnant women who were hospitalized but also all outpatient COVID-19-positive pregnant women. In addition, unlike many studies in the literature, variables such as the educational statuses of infected pregnant women, the effects of the educational status and the gestational age on the risk of getting infected, anticoagulant rates, and medication use rates were also investigated. The limitation of our study is that it is a retrospective, non-randomized study with a small sample size. However, the date range of the study provides homogeneity in terms of evaluating pregnant women infected with the same variant. In addition, our study evaluates short-term maternal and neonatal outcomes. Also, the possibility that the COVID-19-positive pregnant women in our control group may have had asymptomatic COVID-19 that went undetected is a possible limitation our study shares with many other studies in the literature.

## Conclusions

In this study, we observed that COVID-19 positivity during pregnancy did not adversely affect maternal and neonatal outcomes. Also, the gestational age at which patients were positive for COVID-19 did not negatively affect maternal and neonatal outcomes. However, due to the small number of COVID-19-positive pregnant women observed during the early period of the pandemic and the small sample size, it is insufficient to definitively state the assumption that the infection has a positive or negative effect on pregnant women and newborns. There is a need for more large-scale prospective studies on this subject.
